# Numerical Simulation of Arc and Droplet Behaviors in TIG-MIG Hybrid Welding

**DOI:** 10.3390/ma13204520

**Published:** 2020-10-12

**Authors:** Yu Han, Ji Chen, Haijun Ma, Xinyu Zhao, Chuansong Wu, Jinqiang Gao

**Affiliations:** 1 MOE Key Lab for Liquid-Solid Structure Evolution and Materials Processing, Institute of Materials Joining, Shandong University, Jinan 250061, China; 15553776061@163.com (Y.H.); zxy13074161302@163.com (X.Z.); wucs@sdu.edu.cn (C.W.); Jqg@sdu.edu.cn (J.G.); 2 Department of the Special Equipments Safety Supervision, Shandong Administration for Market Regulation, Jinan 250014, China; hjma123@163.com

**Keywords:** TIG-MIG hybrid welding, numerical simulation, arc behavior, metal transfer

## Abstract

Tungsten inert gas-metal inert gas hybrid welding (TIG-MIG) combines the advantages of tungsten and metal inert gas welding. It can efficiently produce high-quality weld joints that meet modern manufacturing quality and efficiency requirements. Based on heat transfer, fluid dynamics, and electromagnetic theory, a three-dimensional coupled transient model of arc-droplet interactions in TIG-MIG hybrid welding was established. In this study, the temperature field, flow field, electromagnetic force, pressure, and current density parameters were analyzed in the arc space. The results show that introducing TIG welding has a significant impact on MIG welding.

## 1. Introduction

As modern manufacturing has continued to develop, welding quality and efficiency requirements have increased. Many studies have shown that tungsten inert gas-metal inert gas hybrid welding (TIG-MIG) is an effective way to combine the respective advantages of single-MIG and single-TIG methods, including high welding efficiency and easy automation for MIG and easy operation and good quality for TIG. This hybrid welding method also has the ability to compensate for shortcomings related to MIG arc instability and low TIG welding efficiency [1,2,3,4,5]. Thus, the synergic effect of “1 + 1 > 2” appears under appropriate hybrid welding parameters.

To develop TIG-MIG hybrid welding, Yang et al. [6] used PID closed-loop control to regulate interactions between two arcs and develop the thermomechanical balance of the molten pool. It was found that using direct-current electrode-positive mode with both TIG and MIG helped to increase weld bead penetration, while using the direct-current electrode negative mode with both TIG and MIG made cleaning the oxide film from the workpiece and suppressing spatters easier. Kanemaru et al. [7] found that there was more metal vapor in TIG-MIG arcs than in single-MIG arcs. This might change the electrical potential inclination of the arc. Moreover, some of the MIG current flowed directly into the TIG electrode from the MIG wire without flowing through the workpiece. This current-divided flow in the hybrid arcs produced a lower total heating efficiency with TIG-MIG than with the single-TIG or -MIG method, and the life of the tungsten in the two arcs was very short as a high welding current was needed to keep the TIG arc (leading arc) straight. To decrease the current-divided flow in TIG-MIG and increase the lift of tungsten, Ding et al. [8] decreased the total welding current to 134A and successfully jointed ferritic stainless steel and magnesium alloys via the TIG-MIG welding-brazing method. Few pores or spatters appeared because of the stable droplet transfer during this welding-brazing process. However, the extremely low heat input decreased the effectiveness of the TIG-MIG hybrid welding. Zong et al. [9] inclined the TIG torch backwards to decrease the TIG current, as the leading TIG arc would be straightened by the repulsive force of the MIG arc. The distance between TIG and MIG arcs was increased to 5.0 mm to avoid current-divided flow in the TIG-MIG method. Experimental temperatures and fluid flows on weld pool surfaces showed that hybrid arcs tend to encourage high-temperature molten metal to move transversely and decrease the backward velocity of molten metal. All of the results mentioned above indicate that the arc and droplet behaviors are key factors that affect heat and force distributions on the weld pool surface, affect the welding process stability, and affect the weld microstructures [10,11,12]. Therefore, it is important to study the interaction mechanisms among the two arcs and the droplet, as these redistribute arc heat and electromagnetic forces on the workpiece and change the droplet trajectory in the arc.

A numerical simulation can be coupled with a few necessary experiments to effectively reveal the physical mechanisms of welding [13,14]. This is also an economic way to provide the quantitative results for heat and mass transfer in the arc and weld pool, which are barely detected when using only experiments. Moreover, building a simple and reliable simulation model is one of the key issues in implementing digital twin technology in the manufacturing industries [15]. Mishima et al. [16] developed three-dimensional TIG-MIG numerical models to study the effects of torch angles on two arcs behavior. The arc plasma temperature changed with the included angle of the two arcs. The lowest heat input occurred when the TIG torch was at 0° and the MIG torch was at 60°. However, the influence of droplet transfer on the arc behavior remains to be studied. Zhou et al. [17] utilized a Gauss heat source model to analyze the heat transfer process during double-sided TIG-MIG processes. It was easier to achieve full penetration using these processes than with the single-MIG method, as the TIG arc increased the MIG arc heating effect during double-sided welding. Chen et al. [18] developed adaptive models of hybrid TIG-MIG arcs to predict the effects of inclined arcs on the workpiece temperature distribution and weld bead geometry. When the distance between the two arc centers was 10.0 mm on the workpiece, heat from the trailing TIG arc did not change the weld pool width because the TIG current was only 125 A. The studies by Zhou and Chen et al. were done to develop the classic heat source models, which could not provide the quantitative distribution of the current density, fluid flow, and electromagnetic force in the two arcs. As a result, the coupling mechanism of the two arcs still needed to be studied. Lou et al. [19] simulated workpiece temperature fields at various electrode distances. The best distance between TIG and MIG electrodes was 10.0 mm because of the preheating effect of the leading TIG arc. The weld pool could be separated into two parts when the electrode distance exceeded 12.0 mm. At this point, the synergistic effect of using two arcs on the workpiece disappeared. In fact, the attractive or repulsive electromagnetic forces produced during TIG-MIG processes not only change arc plasma behavior [20], but also affect droplet behavior. As a result, heat and mass transfer in the weld pool change dramatically, thus influencing the welding process and bead formation [21].

In this study, a three-dimensional transient numerical model of TIG-MIG hybrid arc and droplet dynamic behavior was developed. The temperature, current density, electromagnetic force, pressure, and fluid flow distributions in the arc and droplet were studied and compared to those from the single-MIG method. The influence of arc interactions on arc and droplet behavior was analyzed to reveal TIG-MIG hybrid welding mechanisms. Simulation results were verified using experimental data that addressed the droplet size, droplet tilt angle, and arc tilt angle.

## 2. Mathematical Model

In TIG-MIG hybrid welding, the trailing MIG torch is placed perpendicular to the workpiece, while the leading TIG torch is placed forward and inclined at a particular angle. The following assumptions were made when calculating the hybrid arc: (1) the arc plasma was in local thermal equilibrium (LTE) and optically thin; (2) the arc plasma flow was laminar; (3) the complex physical mechanism of the sheath layer between the cathode and arc plasma or anode and arc plasma was solved via the LTE diffusion approximation method; (4) the workpiece was always flat and heated, and mass transfer within the workpiece could be ignored; (5) the droplet temperature was assumed to be 1800 K when it flowed out of the wire tip; (6) the tungsten tip was flat, with a diameter of 1.0 mm (*D*_Tip_); (7) the droplet behavior was calculated after the arc reached a quasi-steady state.

Based on the above hypotheses, the governing equations for the arc and droplet can be described as follows using the mass continuity equation:

(1) $$\frac{\partial \left(\rho u\right)}{\partial x}+\frac{\partial \left(\rho v\right)}{\partial y}+\frac{\partial \left(\rho w\right)}{\partial z}=0$$

where ρ is the density of the arc plasma or droplet; *u*, *v*, and *w* are the velocity values of the arc plasma or droplet along the *x*, *y*, and *z* axes, respectively.

Momentum conservation equation:

(2) $$\begin{array}{l} \rho \left(\frac{\partial u}{\partial t}+u\frac{\partial u}{\partial x}+v\frac{\partial u}{\partial y}+w\frac{\partial u}{\partial z}\right)=-\frac{\partial p}{\partial x}+\mu (\frac{\partial^{2}u}{\partial x^{2}}+\frac{\partial^{2}u}{\partial y^{2}}+\frac{\partial^{2}u}{\partial z^{2}})+j_{y-TIG}(B_{z-TIG}+B_{z-MIG})\\ -j_{z-TIG}(B_{y-TIG}+B_{y-MIG})+j_{y-MIG}(B_{z-TIG}+B_{z-MIG})-j_{z-MIG}(B_{y-TIG}+B_{y-MIG}) \end{array}$$

(3) $$\begin{array}{l} \rho \left(\frac{\partial u}{\partial t}+u\frac{\partial u}{\partial x}+v\frac{\partial u}{\partial y}+w\frac{\partial u}{\partial z}\right)=-\frac{\partial p}{\partial y}+\mu (\frac{\partial^{2}v}{\partial x^{2}}+\frac{\partial^{2}v}{\partial y^{2}}+\frac{\partial^{2}v}{\partial z^{2}})+j_{z-TIG}(B_{x-TIG}+B_{x-MIG})\\ -j_{x-TIG}(B_{z-TIG}+B_{z-MIG})+j_{z-MIG}(B_{x-TIG}+B_{x-MIG})-j_{x-MIG}(B_{z-TIG}+B_{z-MIG}) \end{array}$$

(4) $$\begin{array}{l} \rho \left(\frac{\partial u}{\partial t}+u\frac{\partial u}{\partial x}+v\frac{\partial u}{\partial y}+w\frac{\partial u}{\partial z}\right)=-\frac{\partial p}{\partial z}+\mu (\frac{\partial^{2}w}{\partial x^{2}}+\frac{\partial^{2}w}{\partial y^{2}}+\frac{\partial^{2}w}{\partial z^{2}})+j_{x-TIG}(B_{y-TIG}+B_{y-MIG})\\ -j_{y-TIG}(B_{x-TIG}+B_{x-MIG})+j_{x-MIG}(B_{y-TIG}+B_{y-MIG})-j_{y-MIG}(B_{x-TIG}+B_{x-MIG}) \end{array}$$

where *p* is the pressure; μ is the dynamic viscosity of the arc or droplet; $j_{x-TIG}$, $j_{y-TIG}$, $j_{z-TIG}$, $j_{x-MIG}$, $j_{y-MIG}$, and $j_{z-TIG}$ are the TIG and MIG arc current densities along the *x*, *y*, and *z* axes, respectively; $B_{x-TIG}$, $B_{y-TIG}$, $B_{z-TIG}$, $B_{x-MIG}$, $B_{y-MIG}$, and $B_{z-MIG}$ are the magnetic flux densities in the TIG and MIG arcs along the *x*, *y*, and *z* axes, respectively.

Energy equation:

(5) $$\begin{array}{l} \rho c_{p}\left(\frac{\partial T}{\partial t}+u\frac{\partial T}{\partial x}+v\frac{\partial T}{\partial y}+w\frac{\partial T}{\partial z}\right)=\frac{\partial }{\partial x}\left(k\frac{\partial T}{\partial x}\right)+\frac{\partial }{\partial y}\left(k\frac{\partial T}{\partial y}\right)+\frac{\partial }{\partial z}\left(k\frac{\partial T}{\partial z}\right)\\ +\frac{j_{x-MIG}^{2}+j_{y-MIG}^{2}+j_{z-MIG}^{2}}{\sigma_{e}}-S_{R-MIG}+\frac{5k_{B}}{2e}(j_{x-MIG}\frac{\partial T}{\partial x}+j_{y-MIG}\frac{\partial T}{\partial y}+j_{z-MIG}\frac{\partial T}{\partial z})\\ +\frac{j_{x-TIG}^{2}+j_{y-TIG}^{2}+j_{z-TIG}^{2}}{\sigma_{e}}-S_{R-TIG}+\frac{5k_{B}}{2e}(j_{x-TIG}\frac{\partial T}{\partial x}+j_{y-TIG}\frac{\partial T}{\partial y}+j_{z-TIG}\frac{\partial T}{\partial z}) \end{array}$$

where *T* is the temperature, $c_{p}$ is the specific heat capacity, *k* is the thermal conductivity, *k_B_* is the Boltzmann constant, *e* is the electron power, $\sigma_{e}$ is the conductivity, and $S_{R-MIG}$ and $S_{R-TIG}$ are radiative heat dissipation of the TIG and MIG arcs, respectively. Clearly, the joule heat, radiation loss, and electron transfer heat of the two arcs are considered in this calculation. The radiation heat dissipation formula is:

(6) $$S_{R-MIG}/S_{R-TIG}=-\varepsilon \zeta (T^{4}-T_{0}^{4})$$

where ε is the emissivity of the arc, ζ is the Boltzmann constant, and *T*_0_ is the ambient temperature of 400 K.

**Figure 1:**
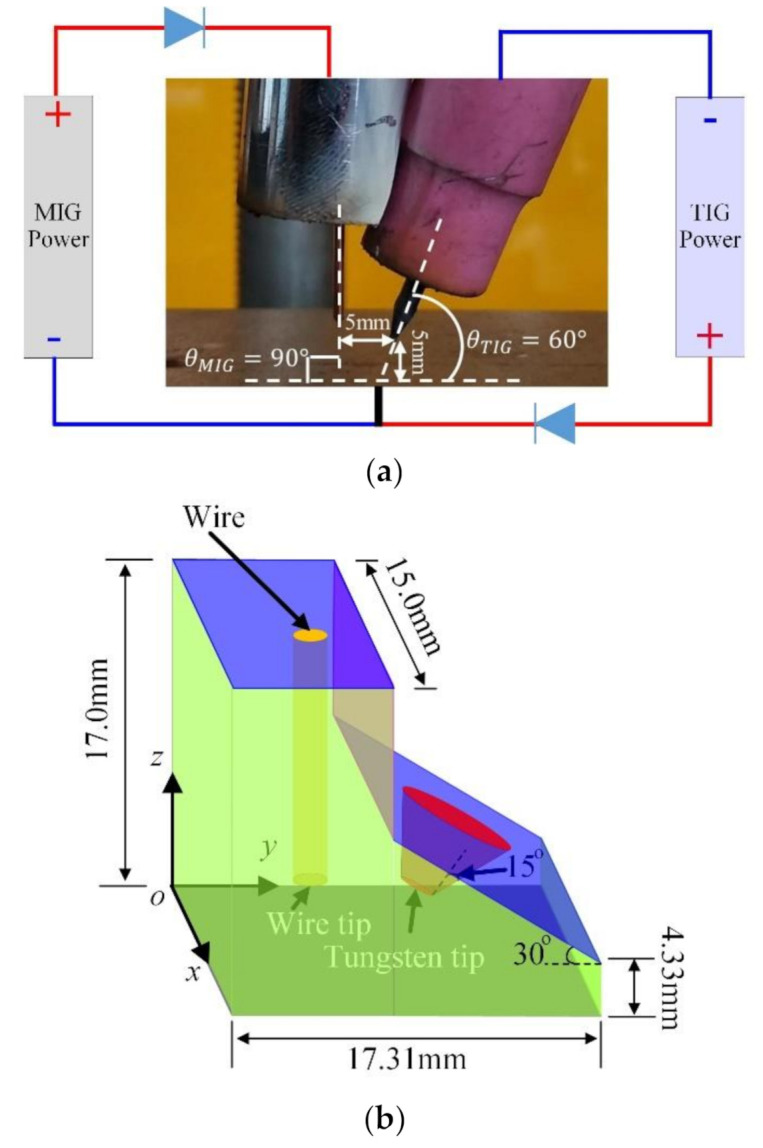
Schematic diagrams of the tungsten inert gas-metal inert gas hybrid welding (TIG-MIG) system. (**a**) Spatial positions of TIG and MIG torches in the experimental system. (**b**) Simplified calculation domain.

If the integration model of the Maxwell equations is adopted to simulate TIG-MIG arcs, most of the welding current flies directly from the positive MIG electrode to the negative tungsten electrode, as the voltage difference between the TIG and MIG electrodes is not only much higher than that between the TIG electrode and the workpiece but also much higher than that between the MIG electrode and the workpiece. Thus, the integration model cannot identify two sets of independent circuit systems, as shown in Figure 1a. In this paper, two groups of current continuity equations and Maxwell equations are established to calculate the coupled effects of the two arcs.

Current continuity equation:

(7) $$\nabla \cdot (\sigma \nabla \phi_{TIG})=0$$

(8) $$\nabla \cdot (\sigma \nabla \phi_{MIG})=0$$

Ohm’s Law:

(9) $$j_{x-TIG}=-\sigma \frac{\partial \phi }{\partial x},\;j_{y-TIG}=-\sigma \frac{\partial \phi }{\partial y},\;j_{z-TIG}=-\sigma \frac{\partial \phi }{\partial z}$$

(10) $$j_{x-MIG}=-\sigma \frac{\partial \phi }{\partial x},\;j_{y-MIG}=-\sigma \frac{\partial \phi }{\partial y},\;j_{z-MIG}=-\sigma \frac{\partial \phi }{\partial z}$$

Maxwell’s equation:

(11) $$\nabla^{2}{\vec{A}}_{TIG}=-\mu_{0}{\vec{j}}_{TIG}$$

(12) $$\nabla^{2}{\vec{A}}_{MIG}=-\mu_{0}{\vec{j}}_{MIG}$$

(13) $$\nabla \times {\vec{A}}_{TIG}={\vec{B}}_{TIG}$$

(14) $$\nabla \times {\vec{A}}_{MIG}={\vec{B}}_{MIG}$$

where $\phi_{TIG}$ and $\phi_{MIG}$ are the electrical potentials in the TIG and MIG arcs, respectively; σ is the electrical conductivity; $\mu_{0}$ is the permeability of vacuum; ${\vec{A}}_{TIG}$ and ${\vec{A}}_{MIG}$ are the TIG and MIG arc magnetization vectors, respectively; and ${\vec{j}}_{TIG}$ and ${\vec{j}}_{MIG}$ are the current vectors, which can be divided into $j_{x-TIG}$, $j_{y-TIG}$, $j_{z-TIG}$, $j_{x-MIG}$, $j_{y-MIG}$, and $j_{z-MIG}$ along the *x*, *y*, and *z* axes, respectively. The magnetic intensity vector ${\vec{B}}_{TIG}$ is composed of $B_{x-TIG}$, $B_{y-TIG}$, and $B_{z-TIG}$, while the magnetic intensity vector ${\vec{B}}_{MIG}$ is composed of $B_{x-MIG}$, $B_{y-MIG}$, and $B_{z-MIG}$.

The volume of the fluid equation is:

(15) $$\frac{\partial F}{\partial t}+u\frac{\partial F}{\partial x}+v\frac{\partial F}{\partial y}+w\frac{\partial F}{\partial z}=0$$

where *F* is the fluid volume fraction.

The boundary conditions on the surface of the wire tip are:

(16) j⇀MIG=I⇀MIGπrMIG2

(17) $$V_{droplet}=\alpha \left|{\vec{I}}_{MIG}\right|+\beta l_{e}{\left|{\vec{I}}_{MIG}^{}\right|}^{2}$$

On the surface of a tungsten tip:

(18) j⇀TIG=−I⇀TIGπrTIG2

where ${\vec{I}}_{MIG}$ is the welding current on the tip of the MIG wire along the wire axis direction; ${\vec{I}}_{TIG}$ is the welding current on the tip of tungsten along the tungsten axis direction; the negative symbol “-” in Equation (18) indicates the flow from the workpiece to the tungsten; $r_{MIG}^{}$ and $r_{MIG}^{}$ are the MIG wire and tungsten tip diameters, which are 1.2 and 1.0 mm, respectively; $V_{droplet}$ is the velocity of a droplet when it flows out from the velocity inlet of the wire, which is represented by a red surface in Figure 1b; $l_{e}$ is the wire extension length, which is 12.0 mm in this calculation; and α and β are two constants with values of 3.11 × 10^−4^ m·A^−1^ and 4.63 × 10^−5^ A^−2^·s^−1^, respectively [22,23].

The green surfaces in Figure 1b are all pressure outlet boundaries. The light blue surfaces are the velocity inlet boundaries for the MIG and TIG arc shield gases, which operate with flow rates of 1.8 (20.0 L/min) and 2.3 m/s (15.0 L/min) at each nozzle tip, respectively. The ceramic TIG nozzle that adjoins the MIG nozzle in Figure 1a is simplified as a purple surface in Figure 1b and indicates a wall in the simulation. The grey face in Figure 1b is the workpiece surface. Other details of the boundary conditions are shown in Table 1.

The droplet transfer was calculated based on a heat and force analysis, which included the joule heat of the arc, the electron enthalpy transport as described by Equation (5), the temperature-dependent surface tension [24], the electromagnetic forces in Equations (2) and (3), the arc drag force [25], and gravity. The simulation environment, the CPU was an Intel (R) Core (TM) i9-9900KS with 16.0 GB RAM. The software used in this study was Fluent 14.5.

## 3. Material Properties and Welding Parameters

The workpiece was Q235 mild steel, the components of which are shown in Table 2. According to the GB/T14957-1944 criteria, the welding wire used in the calculations was H08Mn_2_SiA. Its temperature-dependent thermal properties (surface tension, specific heat, thermal conductivity, and dynamic viscosity) are discussed in [24,26]. The densities of the wire and droplet were constant at 7800 kg/m^3^. The liquidus temperature was 1770 K and the electrical conductivity was 7.0 × 10^5^ S/m. The shielding gas used for MIG and TIG arcs was always argon. Its temperature-dependent thermal properties, such as its density, specific heat, viscosity, thermal conductivity, electrical conductivity, and radiant emission coefficient, are given in [24,26,27,28].

The spatial positions of TIG and MIG torches and electrode connections are shown in Figure 1a, while the welding parameters for single-MIG and TIG-MIG processes are shown in Table 3.

## 4. Simulation Results and Analysis

### 4.1. Temperature Distribution

**Figure 2:**
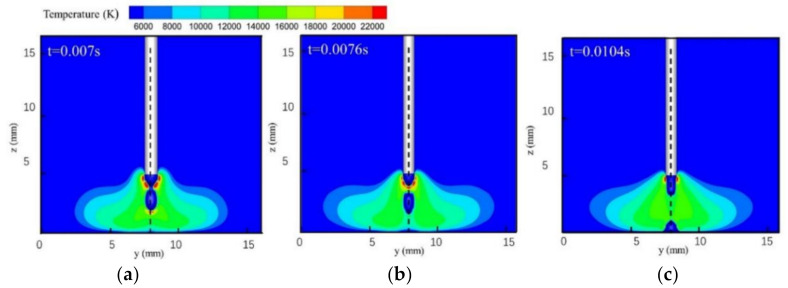
Single-MIG arc temperature distribution: (**a**) *t* = 0.007 s; (**b**) *t* = 0.0076 s; (**c**) *t* = 0.0104 s.

**Figure 3:**
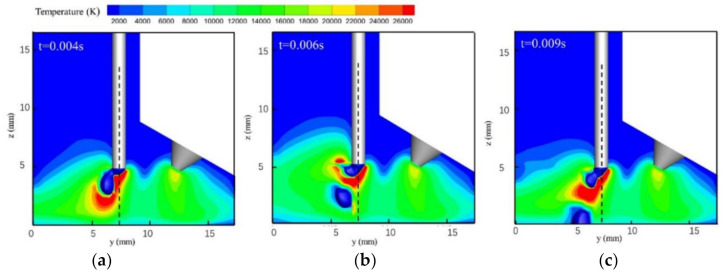
TIG-MIG arc temperature distribution: (**a**) *t* = 0.004 s; (**b**) *t* = 0.006 s; (**c**) *t* = 0.009 s.

Figure 2 and Figure 3 show single-MIG and TIG-MIG arc temperature distributions during droplet transfer. The maximum temperature in the MIG arc is 22,000 K and the typical bell-shaped arc is symmetrical to the axis of the welding wire while the droplet drops from the welding wire tip to the workpiece, as shown in Figure 2. When the TIG arc is introduced into the welding process, Figure 3 shows that the arc incline MIG angle changes from 90° to 113.29°, while the TIG arc incline angle decreases from 60° to 44.38°. This result shows that a repulsive force is produced between the two arcs and that interaction between the two arcs increases the maximum MIG arc temperature from 22,000 to 26,000 K. The trajectory of the droplet movement also changes from 90° to 112.05°, which is quite close to the value for the MIG arc. In the single-MIG welding process shown in Figure 2, the symmetrical arc expands and shrinks periodically during droplet transfer. The maximum arc volume appears when the droplet moves to the center of the arc (z = 2.5 mm). In the TIG-MIG welding process shown in Figure 3, only the left part of the MIG arc (y: 0–5 mm) expands and shrinks periodically during droplet transfer. The TIG arc and right part of the MIG arc are stable when the droplet moves from the wire to the workpiece. This phenomenon indicates that the repulsive force between the two arcs partially restrains the MIG arc. This might be beneficial to the stability of liquid metal in the front weld pool and could encourage this liquid layer to absorb the momentum caused by the droplet impact.

**Figure 4:**
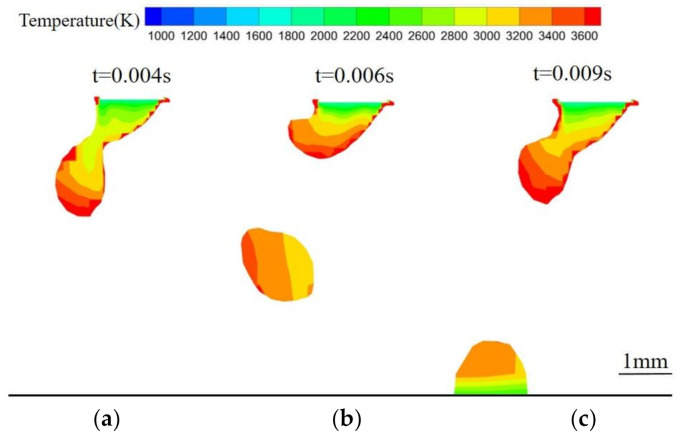
The TIG-MIG droplet temperature distribution: (**a**) *t* = 0.004 s; (**b**) *t* = 0.006 s; (**c**) *t* = 0.009 s.

Figure 4 shows the temperature distribution within a TIG-MIG droplet. The liquid metal at the end of the welding wire melts at 1800 K. The maximum temperature of the droplet reaches 3500 K, while the internal temperature is maintained at approximately 2800 K. After dropping from the wire, the droplet temperature increases slightly, reaching 3200 K. When the droplet reaches the workpiece, its temperature begins to decrease because the current density decreases after away from the end of the wire. The arc temperature also decreases and thermal conduction to the droplet decreases. After the droplet enters the molten pool, its temperature is obviously stratified. The upper layer remains affected by the arc and has a high temperature, while the temperature of the lower layer is lower because of the lower molten pool temperature.

**Figure 5:**
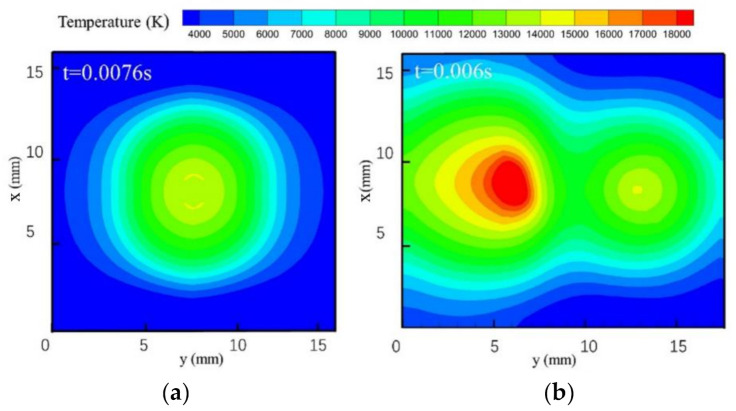
Temperature distribution within the horizontal cross-section at z = 1 mm: (**a**) MIG; (**b**) TIG-MIG.

Figure 5 shows the temperature distributions of TIG and MIG arcs on plane *xoy* near the workpiece (*z* = 1.0 mm). A Gaussian-like temperature distribution field is shown in Figure 5a. The highest temperature (13,000 K, yellow color) appears on a ring with a radius of 2 mm. This high-temperature ring is produced by the droplet as some high-temperature charged particles are hindered from moving from the wire to the workpiece, as shown in Figure 2b. When the TIG arc is introduced into the MIG arc, the temperature field forms the peanut-like shape shown in Figure 5b. A temperature field with two peaks indicates that the repulsive electromagnetic force not only expands the arc action zone on the workpiece, but also increases the heat input from the MIG arc from 13,000 (the single-MIG method) to 20,000 K (TIG-MIG). The maximum temperature of the TIG arc is only 14,000 K near the workpiece. This means that the TIG arc might only preheat the workpiece but not cause melting.

### 4.2. Current Density Distribution

**Figure 6:**
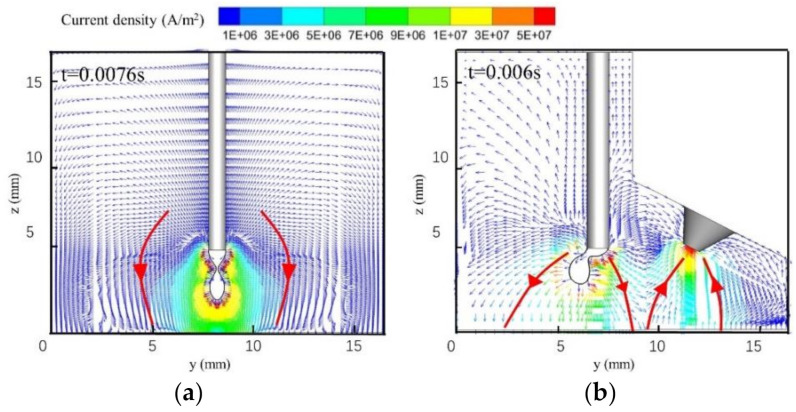
Arc current density distributions: (**a**) MIG; (**b**) TIG-MIG.

Figure 6 shows the current density distributions in the single-MIG and TIG-MIG arcs. The current travels from the end of the welding wire to the workpiece in Figure 6a. The simulated result shows that the single-MIG arc welding current is concentrated around the droplet because of its high electrical conductivity. The current density decreases from 1 × 10^7^ A/m^2^ around the droplet to 7 × 10^6^ A/m^2^ when the droplet arrives at the workpiece. In Figure 6b, the welding current in the MIG arc is no longer symmetrical to the droplet. Most of the MIG current is concentrated on the right side of the droplet and the maximum current density is 3 × 10^7^ A/m^2^. This produces high Joule heat and transport of electron enthalpy. As a result, the right side of the droplet has a higher temperature than the left, as shown in Figure 4. The maximum TIG arc current density is 5 × 10^7^ A/m^2^ and is located near the tip of the tungsten. The relatively low current density area generated between the TIG and MIG arcs is the main reason for the low-temperature region (*y*: 8–11 mm) in Figure 5b. Most of the welding current flows from the wire to the workpiece in the MIG arc and from the workpiece to tungsten in the TIG arc. As shown in Figure 6b, not much of the current flows from the wire to the tungsten. This proves that the hybrid models developed in this manuscript are reasonable.

**Figure 7:**
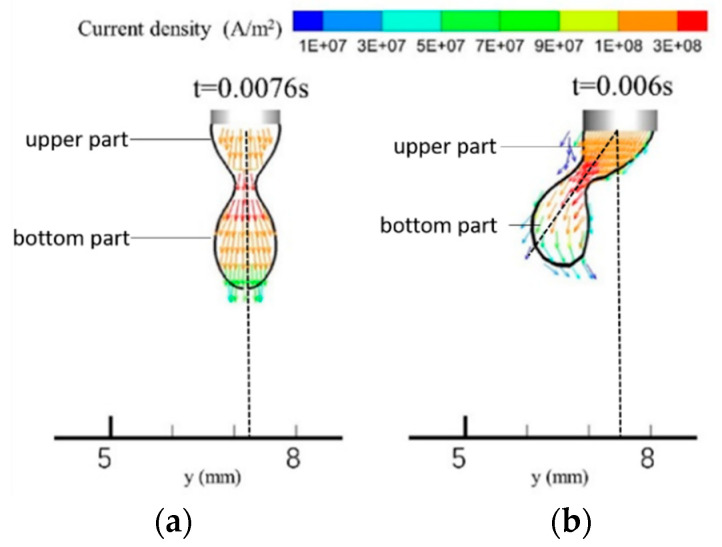
Current density distribution in the droplet: (**a**) MIG; (**b**) TIG-MIG.

Figure 7 shows the welding current density distributions in the single-MIG and TIG-MIG droplets. The maximum current density appears at the droplet necking location in both cases. It appears that the current flows along the direction of the droplet movement. As a result, the direction of the electromagnetic force produced at the droplet necking location should always be perpendicular to the axis of the droplet, regardless of whether the TIG arc is introduced. The current density in the right part of droplet is higher than its left part with the TIG-MIG method, which indicates that the electromagnetic force is higher on the right side than on the left. As a result, the droplet is pushed leftward, as shown in Figure 7b. Moreover, the current density in the upper part of the droplet is higher under TIG-MIG (2 × 10^8^ A/m^2^) than under the single-MIG method (1 × 10^8^ A/m^2^). However, the bottom part of the droplet has a lower current density under TIG-MIG (9 × 10^7^ A/m^2^) than under the single-MIG method (1 × 10^8^ A/m^2^). It is clear that the different current density distributions in the droplet can change the droplet transfer behavior. This will be discussed later.

### 4.3. Electromagnetic Force Distribution

**Figure 8:**
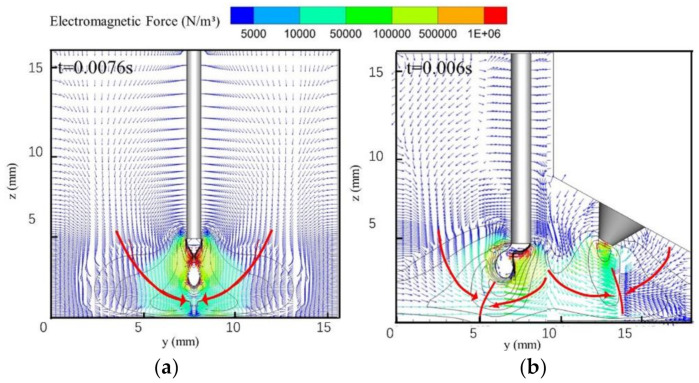
Distribution of electromagnetic force in the arc: (**a**) MIG; (**b**) TIG-MIG.

Figure 8 shows a vector diagram of the electromagnetic force distribution in the arc during droplet necking. The symmetrical electromagnetic force in the single-MIG arc points from the arc edge to its center and tends to restrict the arc shape. The maximum electromagnetic force appears at the droplet necking position and has a value of 1 × 10^6^ N/m^3^ in the single-MIG arc. This value increases to 1.5 × 10^6^ N/m^3^ and the force is located primarily on the right side of the droplet with the TIG-MIG method, as shown in Figure 8b. As a result, the major effect of the electromagnetic force located at the droplet necking location changes from pinching the droplet in the single-MIG method to pushing the upper part of the droplet in the TIG-MIG method, such that the droplet inclines leftward. The incline angle is lower at the bottom part of the droplet (115°) than at the upper part (145°). This indicates that the effect of gravity on the droplet should also be studied.

**Figure 9:**
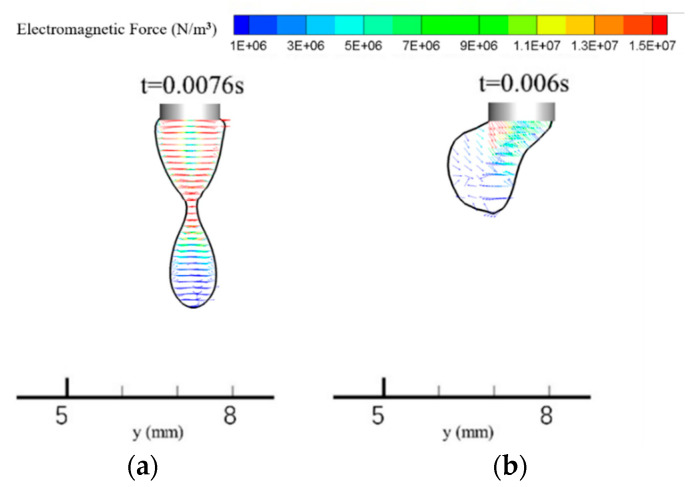
The electromagnetic force distribution in the droplet: (**a**) MIG; (**b**) TIG-MIG.

Figure 9 shows electromagnetic force distributions in single-MIG and TIG-MIG droplets. It is clear that the electromagnetic force is stronger within the droplet than in the arc. The maximum electromagnetic force in the droplet is 1.5 × 10^7^ N/m^3^ and the force is typically stronger in the upper part than in the bottom part. The electromagnetic force decreases when a TIG arc is introduced, whereby its maximum then becomes 1.3 × 10^7^ N/m^3^. This indicates that the electromagnetic force in the arc causes a more significant impact on the TIG-MIG droplet transition than the force in the droplet.

### 4.4. Fluid Flow in the Arc and Droplet

**Figure 10:**
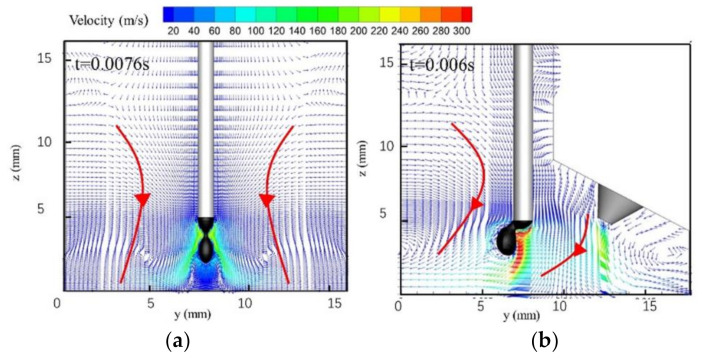
The arc velocity distribution: (**a**) MIG; (**b**) TIG-MIG.

Figure 10 is a vector diagram of the flow field distribution in the arc when the droplet is necked. The fluid flow pattern is shown by the bold red line with the red arrow in its middle. In the single-MIG method, the maximum plasma velocity is approximately 200 m/s. This increases to 300 m/s when the TIG arc is added. The lowest single-MIG arc plasma velocity appears primarily under the droplet and has an average value of 20 m/s. It appears that the droplet blocks the arc plasma fluid flow in the single-MIG method. TIG-MIG arc synergism causes the MIG arc to flow leftward. The arc plasma velocity under the droplet is also higher in the TIG arc than under the single-MIG method. This occurs even though the two arcs merge with each other mainly between the two electrodes (y: 8–13 mm). The maximum hybrid arc velocity appears near the MIG wire axis, which is near the boundary between the two arcs. Based on the electromagnetic force distribution in Figure 8b, the hybrid arc fluid flow is not only dominated by the current density and magnetic intensity. It is also governed by the thermal properties, which depend on the temperature. This indicates that both the electromagnetic force in Figure 8b and the drag force derived from the arc plasma fluid flow push the TIG-MIG droplet to incline leftward.

**Figure 11:**
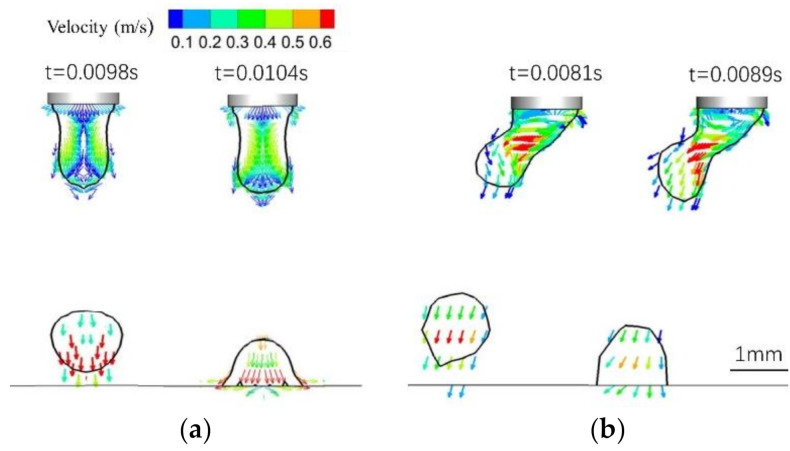
The velocity distribution in the droplet: (**a**) MIG; (**b**) TIG-MIG.

Figure 11 is a vector diagram of the velocity distribution in the droplet. In the single-MIG context, most fluid in the droplet flow converges from the wire top to the droplet axis (necking location) and then diffuses to the bottom of the droplet. The maximum fluid flow appears when the droplet moves near the workpiece. Figure 11a shows a flow field distribution diagram of a single-MIG droplet. In the droplet at the end of the wire, the plasma moves vertically downwards and is squeezed by the electromagnetic force at the droplet neck. The maximum velocity is 0.5 m/s. When the droplet is disconnected, the fluid moves vertically downward within the arc at a maximum velocity of 0.6 m/s. After the droplet moves to the workpiece, the plasma direction changes to along the workpiece. This can encourage forward and backward metal flow in the molten pool. For the TIG-MIG method, the direction of the fluid flow in the droplet is consistent with the direction of the droplet deflection. The maximum speed of 0.6 m/s is observed near the droplet necking position. It is clear that this type of droplet movement helps to accelerate heat transfer from the arc to the droplet. When the droplet reaches the workpiece, most molten metal in the droplet moves leftward and downward. Comparing Figure 11a with Figure 11b shows that downward momentum of the TIG-MIG droplet is decreased by the repulsive force between the two arcs and that the leftward speed of the droplet increases to 0.6 m/s. The reduced vertical momentum helps to improve the thickness of the liquid layer under the arc and increases its ability to absorb the droplet momentum. However, a receding droplet increases the backward liquid flow velocity and increases the risk of welding defects. This combined effect should be analyzed further.

### 4.5. The Droplet Transition

**Figure 12:**
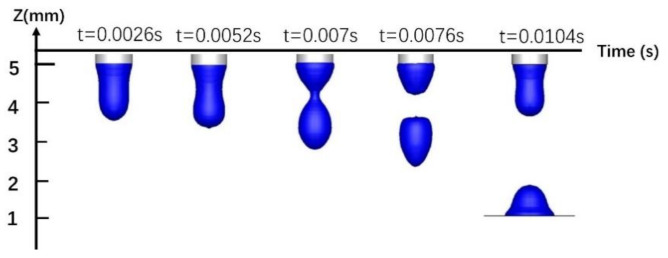
Single-MIG welding droplet transition.

**Figure 13:**
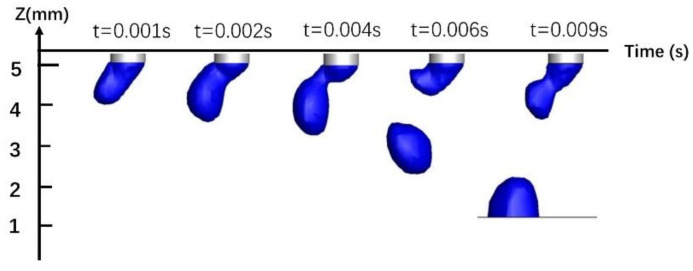
TIG-MIG composite welding droplet transition.

Due to the combined effects of surface tension, electromagnetic force, gravity, and plasma flow, the droplets first accumulate and grow at the end of the wire, then neck down and finally drop onto the workpiece. Figure 12 shows a single-MIG droplet transition. The droplet is subject to self-induced electromagnetic force, which is applied uniformly to its left and right sides. After the droplet is broken, it falls vertically into the molten pool along the welding wire axis. The droplet transition period is 0.0104 s. Figure 13 shows the transition pattern of a TIG-MIG composite arc droplet. In addition to the self-induced electromagnetic force, the droplet is affected by the TIG Lorentz force. The Lorentz force of the TIG arc on the MIG arc is quite small compared to the self-induced electromagnetic force. The effect of the TIG Lorentz force on the droplet transfer frequency is negligible, but it has a large effect on the droplet deflection. At 0.004 s, contraction of the self-induced electromagnetic force causes the droplet to neck. After entering the molten pool, the second droplet continues to grow at the end of the wire and the droplet transition period is 0.009 s. The droplet transition period is shorter than with single-MIG droplet transfer. This is related to the electromagnetic and plasma flow forces discussed in the previous analysis. The plasma flow force around the droplet is larger during hybrid welding than during single-MIG welding. This promotes droplet transition.

## 5. Experimental Validation

In order to validate the simulation results, experiments were conducted using a welding speed of 1 m/min. Images were collected using high-speed cameras to observe metal transfer. In addition, the deflection angles of arcs and droplets were measured.

**Figure 14:**
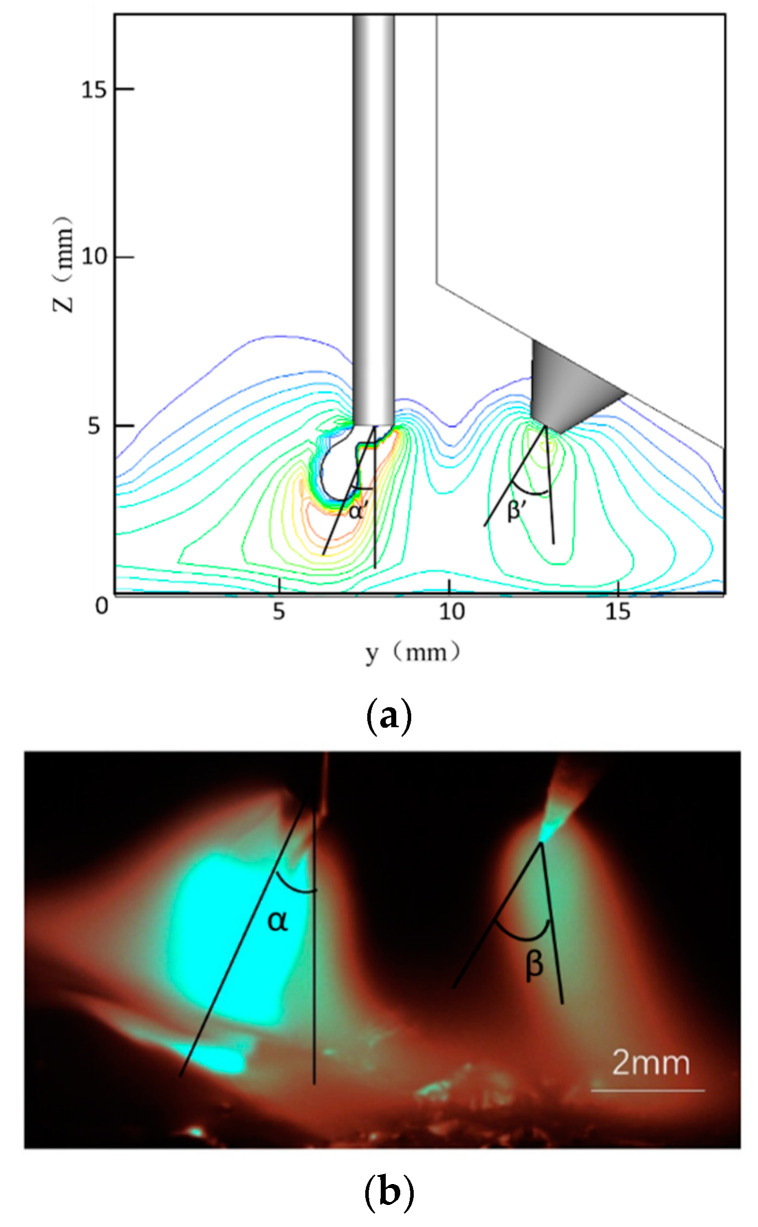
A comparison of MIG and TIG arc deflections: (**a**) arc shape simulation; (**b**) experimental diagram of the arc shape.

Figure 14 compares MIG and TIG arc deflection. The MIG arc deflects to the left while the TIG arc deflects to the right. The simulated MIG arc angle (α’) is 22°, while that of the TIG arc (β’) is 37°. The experimental result produces a MIG arc angle (α) of 25° and TIG arc angle (β) of 39°.

**Figure 15:**
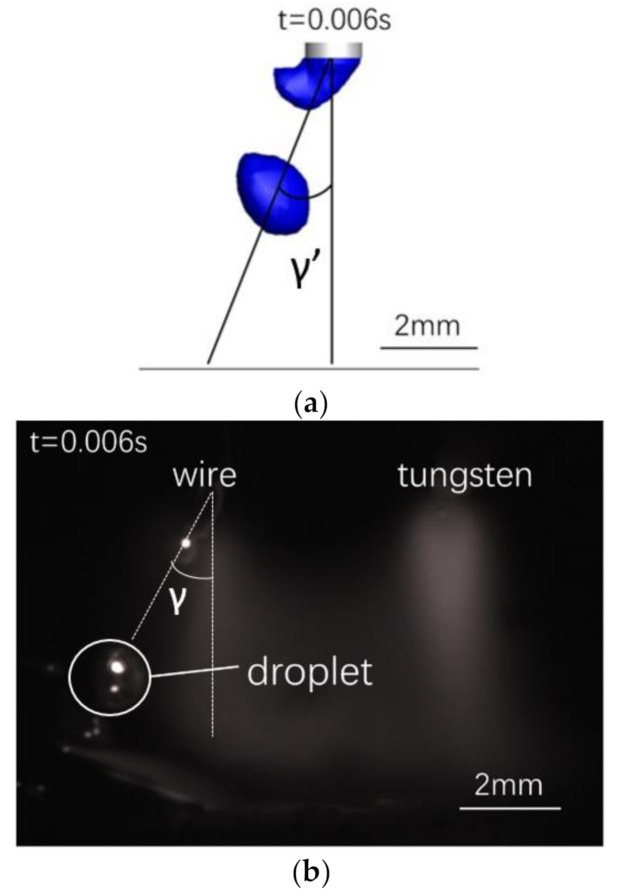
Comparison of (**a**) simulated and (**b**) experimentally observed droplets.

A simulation of a droplet at 0.006 s (Figure 15a) is compared to experimental data from a droplet at the same time in one transition period (Figure 15b). The droplet deflection is 28° in the simulation result (γ’) and 24° in the experimental result (γ). The droplet diameter is 1.2 mm in both the simulation and experiment. Thus, the experimental and simulation results are in good agreement.

The calculated and experimental results are within a reasonable error range. This indicates that the three-dimensional TIG-MIG composite arc–droplet model used in this study is correct and the simulation results are accurate.

## 6. Conclusions

In order to explore and clarify TIG-MIG hybrid arc–droplet interaction mechanisms and the effect of the arc droplet on the workpiece, this study combined numerical simulation with experimental methods to compare and analyze single-MIG and TIG-MIG hybrid arc–droplet interactions, as well as heat and force distributions on the workpiece. The following conclusions were drawn:

- The arc shapes from single-MIG and TIG-MIG hybrid welding were significantly different. Interactions between hybrid arcs changed the plasma flow direction and velocity, causing the two arcs to deflect from the axis in opposite directions and producing a camel hump shape;
- Changes in the hybrid arc shape changed the distribution of heat from the arc to the workpiece. The heat source distribution elongated along the weld direction, while the heat source range decreased in the vertical weld direction;
- During the process of metal transfer, the arc shape changed constantly. The electromagnetic force, current density, and the flow field were no longer concentrated at the end of the wire. Instead, they were distributed around the droplet. This affected the temperature distribution of the arc on the workpiece and the workpiece shape;
- Droplets from MIG and TIG-MIG welding were compared. The additional horizontal velocity associated with the TIG-MIG droplet pushed the droplet to the left. The direction of flow into the molten pool was no longer vertically downward. Instead, it was along the direction of the inclined droplet and served to push the metal flow.

## Figures and Tables

**Table 1:** Boundary conditions.

Boundary	*T* (K)	$\sqrt{u^{2}+v^{2}+w^{2}}\;(m/s)$	ϕ (V)	$\vec{A}$ (T m)
Velocity inlet of MIG shielding gas	400.0	1.8	$\frac{\partial \phi }{\partial \vec{n}}$	$\frac{\partial \vec{A}}{\partial \vec{n}}$
Velocity inlet of TIG shielding gas	400.0	2.3	$\frac{\partial \phi }{\partial \vec{n}}$	$\frac{\partial \vec{A}}{\partial \vec{n}}$
Pressure outlet boundaries	1000.0	–	$\frac{\partial \phi }{\partial \vec{n}}$	0
Wire tip surface	1800.0	$V_{droplet}$	${\vec{j}}_{MIG}$	$\frac{\partial \vec{A}}{\partial \vec{n}}$
Tungsten tip surface	2600.0	0.0	${\vec{j}}_{TIG}$	$\frac{\partial \vec{A}}{\partial \vec{n}}$
Workpiece surface	2000.0	0.0	0.0	$\frac{\partial \vec{A}}{\partial \vec{n}}$

**Table 2:** Components for Q235 and H08Mn2SiA.

Workpiece and Wire	C	Mn	Si	P	S
Q235	0.12	0.50	0.25	0.015	0.035
H08Mn2SiA	0.08	1.95	0.78	0.011	0.014

**Table 3:** Welding parameters used in single-MIG and TIG-MIG simulations.

Welding Method	Welding Current (A)	Arc Voltage (V)	Thermal Emissivity (%)	Torch Angles (°)	Shielding Gas Flow Rate (L/min)
MIG	250.0	30.0	80.0	90.0	20.0
TIG-MIG	100.0–250.0	11.7–30.0	80.0–80.0	60.0–90.0	15.0–20.0
